# Effect of national COVID-19 lockdown on the incidence of muscle, tendon and ligament injuries and related surgical procedures in the working-aged Finnish population

**DOI:** 10.1007/s00402-022-04521-2

**Published:** 2022-07-02

**Authors:** Julius Möttönen, Ilari Kuitunen, Mikko Uimonen, Ville M. Mattila, Juha Paloneva, Ville Ponkilainen

**Affiliations:** 1 School of Medicine, University of Eastern Finland, Yliopistonranta 1, 70211 Kuopio, Finland; 2 Mikkeli Central Hospital, Porrassalmenkatu 35-37, 50100 Mikkeli, Finland; 3 Department of Surgery, Hospital Nova of Central Finland, Keskussairaalantie 19, 40620 Jyväskylä, Finland; 4 Department of Orthopaedics and Traumatology, Tampere University Hospital, Teiskontie 35, PL2000, 33521 Tampere, Finland; 5 Faculty of Medicine and Health Technology, Tampere University, Tampere, Finland; 6 COXA Hospital for Joint Replacement, Biokatu 6, 33520 Tampere, Finland

**Keywords:** COVID-19, Lockdown, Trauma, Surgery, Incidence

## Abstract

**Introduction:**

The effect of lockdown on the incidence of fractures and severe injuries has been widely studied, whereas studies regarding muscle, tendon, and ligament injuries have not received as much attention. The aim of the study was to investigate the effect of the lockdown and later regional regulations due to the COVID pandemic on the incidence of muscle, tendon, and ligament injuries and related surgical procedures.

**Materials and methods:**

This study focuses on the working-age population in the catchment areas of three major Finnish hospitals. Patients were divided into three age groups 18–34, 35–50 and 51–65 years of age. Suitable injuries were retrieved from the data using appropriate ICD-10 codes and procedure codes. The monthly incidence rate ratio (IRR), with 95% confidence intervals (CI), were compared between the year 2020 and the reference years 2017–2019.

**Results:**

Upper and lower extremity injury ED visits decreased by 15.7 and 8.2%. For upper extremity injuries, a decrease in incidence was observed for all three age groups in March (IRR 0.52, CI 0.33–0.80), (IRR 0.53, CI 0.31–0.91), (IRR 0.60, CI 0.38–0.95), respectively. An increase in 18–34 years of age group was detected in June (IRR 1.49, CI 1.05–2.13). Lower extremity injuries decreased in 18–34 years of age group in March (IRR 0.62, CI 0.43–0.90) and April (IRR 0.60, CI 0.42–0.87).

A decrease on the incidence of surgeries was observed in April for the 35–50 (IRR 0.53, CI 0.29–0.97) and 51–65 years of age groups (IRR 0.58, CI 0.34–0.98).

**Conclusions:**

The nationwide lockdown in spring 2020 led to a notable decrease in the incidence of emergency department visits and the surgical treatment of muscle, tendon, and ligament injuries in Finland.

**Supplementary Information:**

The online version contains supplementary material available at 10.1007/s00402-022-04521-2.

## Introduction

The COVID-19 outbreak led to many countries declaring national lockdowns to prevent the further spread of the virus [1]. On March 16, 2020, the Finnish government announced a state of emergency and implemented a nation-wide lockdown in response to the outbreak. People were ordered to limit their daily movements by working from home, and hobbies and sporting events were suspended. In addition, social contacts were minimized, and schools and other educational institutions, nightclubs, and restaurants were closed. In health care, many non-urgent visits and surgeries were postponed or canceled, and it was recommended that citizens should not seek help from the emergency department (ED) unless it was absolutely necessary [2]. In the fall of 2020, Finland faced a second COVID-19 outbreak. Instead of implementing a second national lockdown, however, the Finnish government implemented regional restrictions. These regional restrictions included a more restrained stepwise approach that partly allowed hobbies and sport events to continue and nonacute medical procedures to be performed [3].

To date, the effect of the lockdown on incidences of fractures and severe injuries have been widely studied, whereas studies regarding muscle, tendon, and ligament injuries have not gained much attention [4–6]. Previous studies have reported that trauma admissions in the ED decreased during the lockdown [5, 7, 8]. Furthermore, this decrease in admissions has been shown to be oriented towards the younger population [5, 9]. A study from 2021 reported that although trauma admissions decreased as a whole during lockdown, the incidence of severe injuries remained at the same level as before the lockdown [6]. In Finland, pediatric soft tissue injuries decreased significantly during the lockdown in 2020. This decrease was mostly seen in 4–17 years of age group who are at an age when many minor injuries are the result of participating in sports and other hobbies [4]. A study from Italy reported that shoulder and elbow trauma related to sports and daily commuting decreased during the lockdown [10].

In this study, we investigate the incidence of upper and lower extremity injuries and related surgical procedures in the working-age population that occur in those activities restricted by the implementation of nationwide lockdown, such as sporting activities and daily commuting.

## Methods

This retrospective study is based on data collected from three large Finnish hospitals–Central Finland Hospital (CFH), Tampere University Hospital (TAUH), and Mikkeli Central Hospital (MCH). The study focuses on the working-age population of Finland who are generally between 18 and 65 years of age. The total combined catchment population of the three hospitals is 5,30,000 [11]. The data consists of all the visits and surgeries performed at these three hospitals between the reference years 2017–2019 and 2020. Information on visits, diagnoses, surgeries, procedure codes, hospitalization times and whether the surgeries were elective or not, was acquired from the electronic discharge registers of these three hospitals.

In the present study, we focused on the upper and lower extremity injuries that most commonly occur in activities restricted by the implementation of the lockdown, such as sports, daily commuting, hobbies, and other spare time activities. We included ED visits based on the ICD-10 diagnostic code system. For upper extremity injuries, we used codes that describe elbow/forearm, shoulder, wrist and hand dislocations, sprains, muscle injuries, and tendon/ligament injuries. For lower extremity injuries, we used codes that describe knee/shin, ankle/foot, and hip and thigh dislocations, sprains, muscle injuries, and tendon/ligament injuries. Surgeries were retrospectively retrieved from the electronic medical record systems of the participating hospitals using Nordic Medico-Statistical Committee (NOMESCO) Classification of Surgical Procedures (NCSP) procedure codes [12]. For upper and lower extremity surgeries, we selected codes that are used for soft tissue shoulder, elbow, wrist/hand, hip/thigh, knee/shin, and ankle/foot surgeries. We excluded procedure codes that were not remedial by nature or were not typically induced by trauma. A complete list of the included ICD-10 and procedure codes with definitions is provided in Appendix 1.

The aim of the study was to compare the incidence of upper and lower extremity injuries and the injury related surgeries per 1,00,000 persons during the nationwide lockdown from March 16 to June 1, and during the regional restrictions from September to December in 2020 to the mean incidence of upper and lower extremity injuries and the injury related surgeries per 1,00,000 persons during the reference years 2017–2019 focusing on the same calendar periods. The incidence of injuries and surgeries were stratified in the following age groups: 18–34, 35–50, and 51–65 years of age. The age groups were equal in size and were selected to describe age-dependent variation in injury incidence and surgeries. The data were also stratified to upper and lower extremity injuries with the same age distribution.

The monthly incidence per 1,00,000 inhabitants of injuries and surgeries were calculated for the year 2020 and for the reference years 2017–2019 using the mean population size of the combined catchment area of the three hospitals. The incidence rates were compared using the incidence rate ratios (IRRs) with 95% confidence intervals (CI). All the statistical analyses were performed with R version 4.1.0 [13]. According to current Finnish legislation, register-based studies do not require ethics committee approval [14].

## Results

During the follow-up period 2017–2020, there were a of 3,77,997 ED visits and 1,08,708 surgeries in 18–65 years of age group in all three hospitals. The average number of ED visits for the reference years 2017–2019 was 96,122 per year and 27,329 surgeries per year. In 2020, a 6.8% decrease (*n* = 89,631) in ED visits and a 2.2% decrease in surgeries (*n* = 26,720) was observed.

### Upper extremity injuries

**Fig. 1:**
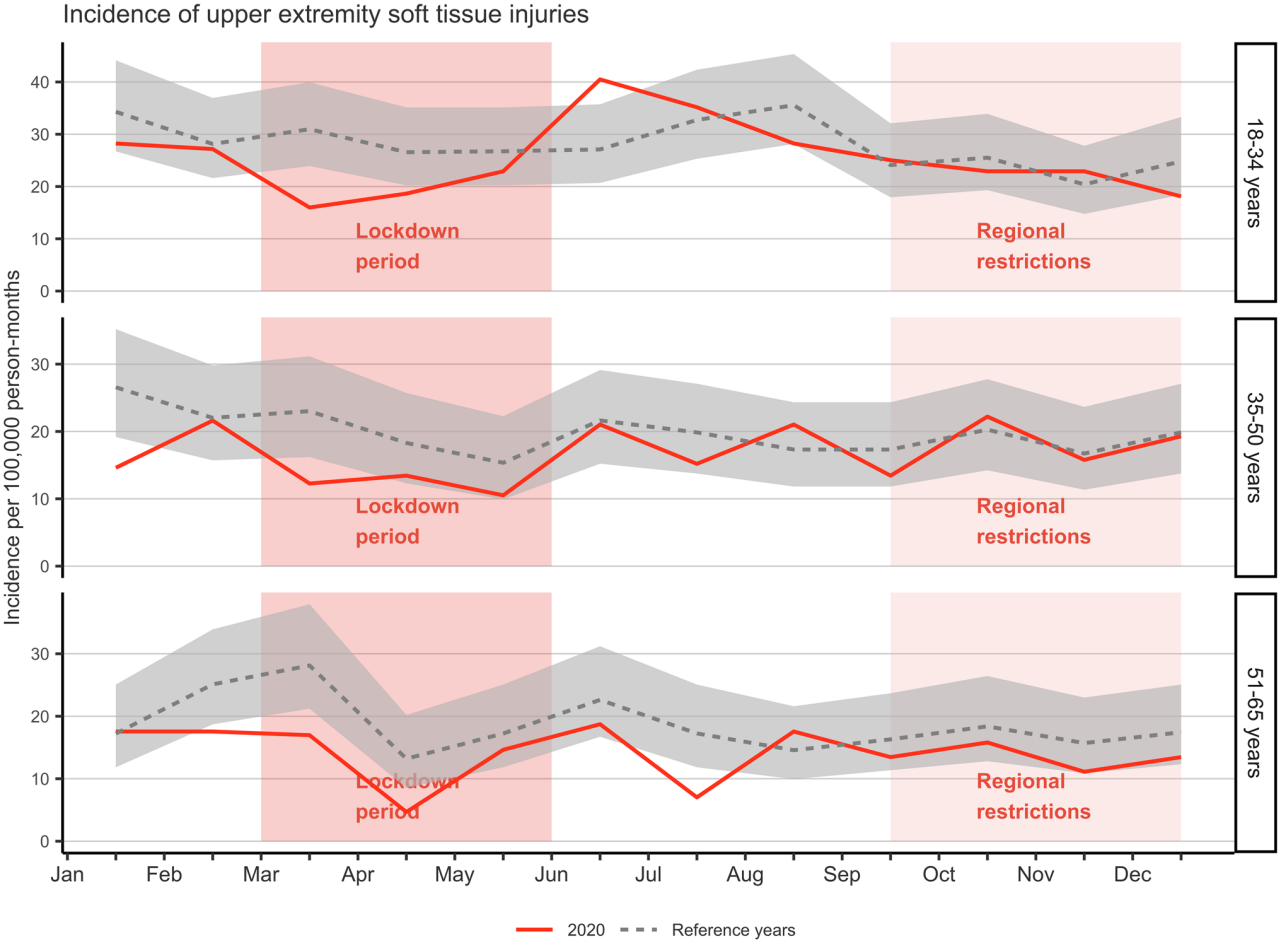
Incidence of upper extremity soft tissue injury emergency department visits in 2020 (solid line) compared to the reference years (2017–2019) (dashed line) with 95% CI (gray area) including the periods of lockdown (March–June) and regional restrictions (September–December)

ED visits due to upper extremity injuries decreased by 15.7% in 2020 compared to the mean of the reference years (*n* = 1205 vs. *n* = 1430). During the early lockdown period in March, a decrease in incidence was observed in all three age groups: 18–34 years of age (IRR 0.52, CI 0.33–0.80), 35–50 years of age (IRR 0.53, CI 0.31–0.91), and 51–65 years of age (IRR 0.60, CI 0.38–0.95) (Fig. 1). In the oldest age group, a decrease in incidence was also observed during the lockdown in April (IRR 0.35, CI 0.16–0.79) and after the lockdown in July (IRR 0.41, CI 0.21–0.80). By contrast, the incidence of upper extremity injuries increased slightly in 18–34 years of age group in June (IRR 1.49, CI 1.05–2.13). However, no remarkable decreases in the incidence of upper extremity injuries were observed during the regional restrictions.

### Lower extremity injuries

**Fig. 2:**
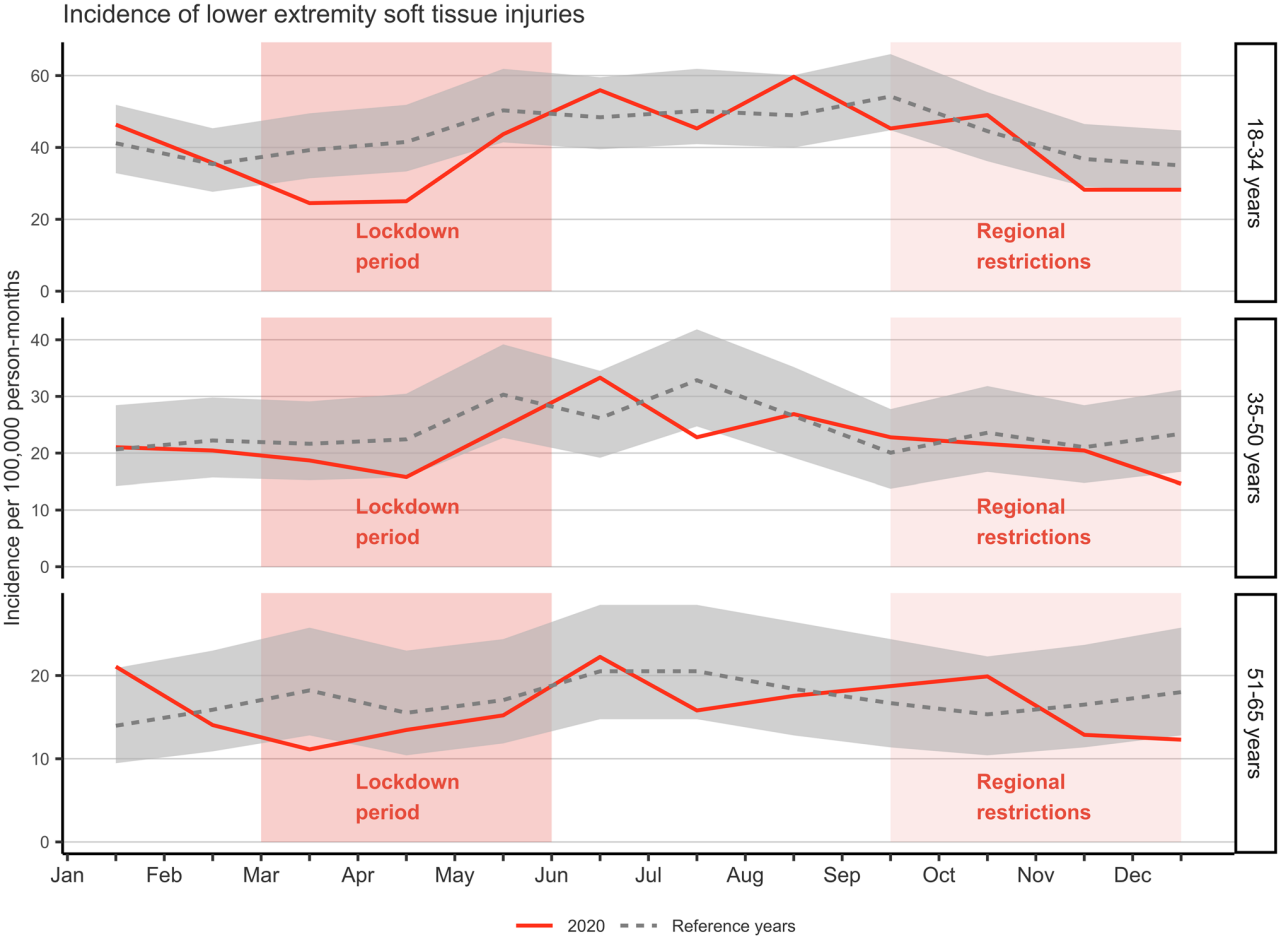
Incidence of lower extremity soft tissue injury emergency department visits in 2020 (solid line) compared to the reference years (2017–2019) (dashed line) with 95% CI (gray area) including the periods of lockdown (March–June) and regional restrictions (September–December)

ED visits due to lower extremity injuries decreased by 8.2% in 2020 compared to the average number of visits in the reference years (*n* = 1696 vs. *n* = 1848). Moreover, during lockdown, the incidence of lower extremity injuries in 18–34 years of age group decreased in March (IRR 0.62, CI 0.43–0.90) and April (IRR 0.60, CI 0.42–0.87) (Fig. 2). No other substantial decreases in the incidence of lower extremity injury visits were observed during lockdown or regional restrictions.

### Surgeries

**Fig. 3:**
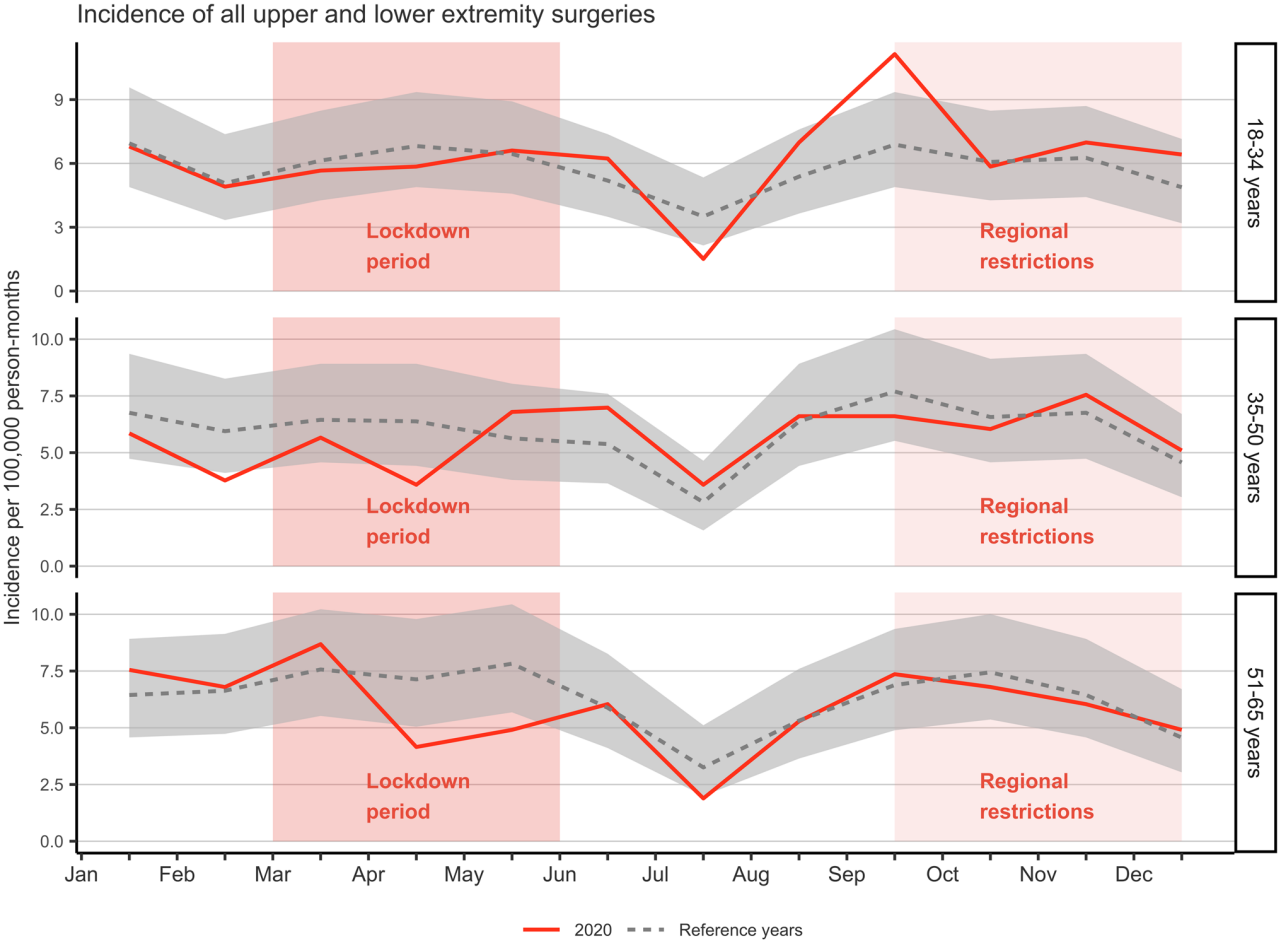
Incidence of all upper and lower extremity surgeries (elective and urgent) in 2020 compared to the reference years (2017–2019) with 95% CI (gray area) including the periods of lockdown (March–June) and regional restrictions (September–December)

The total number of upper and lower extremity surgeries did not considerably differ between 2020 (*n* = 1131) and the average of the reference years 2017–2019 (mean *n* = 1152). A decrease in the incidence of surgeries during the lockdown was observed in April in 35–50 years of age group (IRR 0.53, CI 0.29–0.97) and in 51–65 years of age group (IRR 0.58, CI 0.34–0.98) (Fig. 3). In the youngest, 18–34 years, age group, no decreases in the incidence of surgeries were observed during lockdown. However, there was a rebound in the incidence at the start of the regional restrictions in September (IRR 1.62, CI 1.04–2.52). No decreases in the incidence of surgeries were observed during regional restrictions in any of the age groups.

## Discussion

Incidence of ED visits regarding muscle, tendon, and ligament injuries in both the lower and upper extremities decreased in all age groups and especially in young adults during the COVID-19 lockdown in spring 2020. Decreases in ED visits were, according to our data, not observed during the regional restrictions implemented in response to the second wave of COVID-19. After the lockdown, ED visits and surgeries in the youngest age group rebounded to a level above the annual average of the reference years.

In the present study, we found a decrease in the incidence of upper and lower extremity soft tissue injuries in all three age groups during lockdown. In two Italian studies, the incidence of soft tissue injuries was also found to have decreased, especially in the younger population [10, 15]. However, as the pandemic spread rapidly in Italy and similar lockdown acts were not implemented, they mainly studied the effects of the disease rather than the effects of the lockdown [10, 15]. A previous Finnish study has reported similar results with the same lockdown measures in children [4]. Our results show a decreasing trend in the youngest age group, but in contrast to the Italian studies, and 2020 study done in France, we saw a remarkable decrease in older population as well [5, 10, 15]. It seems, therefore, that the lockdown affected all kinds of injuries, and the effect is seen throughout the age distribution in working aged people. The implementation of lockdown measures, such as the suspension of hobbies and sport events and the reduction in commuting, could explain the more extensive impact on young adults who are typically more mobile and who more actively attend sports and recreational activities compared to older adults.

Injuries of the upper extremity decreased more than lower extremity injuries in our data. In upper extremity injuries, the decrease was found in all three age groups but was the most prominent in the youngest (18–34 years) and the oldest (51–65 years) age groups. A decrease in lower extremity injuries was only seen in young adults. This pre-pandemic epidemiological data from Iranian population shows that upper and lower extremity injuries are mostly caused by a fall, direct hit, sports, or traffic accidents [16, 17]. Therefore, the results suggest that the restrictions in daily movement, the closing of restaurants and shopping malls, and the suspension of hobbies and sports could have affected the incidence of these types of injuries. A study from New Zealand showed that while the total number of injuries and the share of sports- and commuting-related injuries decreased during the lockdown, the share of injuries occurring in the home increased [18]. The difference between age groups may be due to the older population doing more physical work in and around the household, whereas the younger population spend more time doing sports and hobbies. An increase in upper extremity injuries seen in young adults in June could be the consequence of the end of the lockdown when people started to move and exercise more or sought help from the ED more often.

A decrease in the incidence of surgeries due to soft tissue injuries was observed in the older age groups (35–50, 51–65). Surprisingly, we did not see a decrease in the incidence of surgeries in the youngest age group even though this group had the largest decrease in the incidence of ED visits. As the injuries requiring surgery are usually more severe than those treated conservatively, our findings suggest that the decrease in severe injuries was not as prominent as in other injuries. Similar findings have also been found from pediatric trauma surgeries and multiple injury surgeries as well [4, 6]. We studied both elective and nonelective surgeries, and elective surgeries were mostly postponed during the lockdown. This may have caused the slight decreases in the incidence seen in the older groups [19]. In contrast, the incidence of musculoskeletal injury surgeries in France decreased considerably during lockdown [5]. We also saw an increase in the incidence of surgeries at the start of the regional restrictions in September in 18–34 years of age group. In addition, the incidence of ED visits in this same age group rebounded in June. This rebound in the incidence of surgeries could be a consequence of the suspension of elective surgery during the summer in Finland and from the elevated incidence of ED visits after the lockdown.

The resources of the hospital districts are carefully distributed to target the incidence of different types of visits and surgeries [20]. Therefore, more information on how the incidence of muscle, ligament, and tendon injuries and surgeries vary during and after the implementation of nationwide lockdown can improve the reorganization of these resources during times of similar restrictions.

Having access to a large database from three hospitals with a sizable catchment population (5,30,000) and having information on all health care levels (primary, secondary, and tertiary) is a major strength of this study and adds to the general applicability of the findings to the Finnish population. Moreover, as the application of ICD-10 codes and procedure codes after hospital visits or procedures is compulsory in Finland, the database is more reliable. Moreover, we studied ED visits and surgeries occurring in publicly funded hospitals with unified treatment protocols. However, a major weakness of this study is the missing data on injury mechanisms. Without information on injury mechanism, we could not properly discuss the possible factors behind our results. We had only 3 years of the reference data that causes more uncertainty in the representation of the differences between the incidences. Furthermore, the data do not include the visits and surgeries of those people who receive occupational health care or who have used private medical services. In addition, because of the elective nature of these types of soft tissue injury surgeries, we could not confirm whether all the surgeries were the result of the visits presented in our data.

## Conclusions

The incidence and number of upper and lower extremity soft tissue injuries decreased during lockdown in the working-age population, whereas the incidence remained stable during the regional restrictions in 2020. The decrease was the most prominent in upper extremity injuries and in young adults. While we saw a decrease in the incidence of surgeries in upper and lower extremity injuries, it was not as pronounced as the decrease in ED visits. After the lockdown, the incidence of injury visits and surgeries rebounded in the youngest age group. Regional restrictions did not affect to the incidence of surgical treatment of these injuries. The healthcare´s burden on ED visits and surgical treatment, regarding these injuries, eased during the lockdown. These findings can possibly be used in future states of emergencies in the distribution on ED and surgical resources in large hospitals.

## Supplementary Information

Below is the link to the electronic supplementary material.

Supplementary file1 (DOCX 15 KB)
