# Supplementary material for: Effect of national COVID-19 lockdown on the incidence of muscle, tendon and ligament injuries and related surgical procedures in the working-aged Finnish population
Source: Arch Orthop Trauma Surg. 2022 Jul 2;143(5):2539–45. doi: 10.1007/s00402-022-04521-2 (PMC10110679; doi:10.1007/s00402-022-04521-2)
Supplement: Supplementary file 1 — Supplementary file1 (DOCX 15 KB) [file 402_2022_4521_MOESM1_ESM.docx]

**APPENDIX 1.**

**Table 1. Diagnose codes (ICD-10 diagnostic code system).**

| Diagnose code | Definition | Subcodes used |
| --- | --- | --- |
| S43 | Dislocations, sprains and strain of the shoulder joints and ligaments | All |
| S46 | Muscle and tendon injuries of the shoulder | All |
| S53 | Dislocation, sprain and strain of joints and ligaments of elbow | All |
| S56 | Injury of muscle and tendon at forearm level | All |
| S63 | Dislocation, sprain, and strain of the wrist joint and ligaments | All |
| S66 | Muscle and tendon injuries of the wrist and hand | All |
| S73 | Dislocation, sprain and strain of joints and ligaments of hip | All |
| S76 | Injury of muscle and tendon of hip and upper leg level | All |
| S83 | Dislocation, sprain and strain of joints and ligaments of knee | All |
| S86 | Injury of muscle and tendon at lower leg level | All |
| S93 | Dislocation, sprain and strain of joints and ligaments of ankle and foot | All |
| S96 | Muscle and tendon injuries of the ankle and foot | All |

**Table 2. Operation codes (NCSP Finnish version).**

| Operation code | Definition | Subcodes used |
| --- | --- | --- |
| NBA | Exploratory procedures on shoulder and upper arm | All |
| NBE | Operations on capsules and ligaments of joints of shoulder | NBE20, NBE25, NBE30, NBE35, NBE40, NBE45, NBE70, NBE72 |
| NBH | Miscellaneous operations on joints of shoulder (reduction of dislocations) | All |
| NBL | Operations on muscles and tendons of shoulder and upper arm | All |
| NCA | Exploratory procedures of elbow and forearm | All |
| NCE | Operations on capsules and ligaments of elbow joint | NCE20, NCE25, NCE30, NCE35 |
| NCH | Miscellaneous operations on elbow joint (reduction of dislocations) | All |
| NCL | Operations on muscles and tendons of elbow and forearm | All |
| NDA | Exploratory procedures on wrist and hand | All |
| NDE | Operations on capsules and ligaments of joints of wrist and hand | NDE20, NDE25, NDE40, NDE60, NDE62, NDE64, NDE68 |
| NDH | Miscellaneous operations on joints of shoulder (reduction of dislocations) | All |
| NDL | Operations on muscles and tendons of wrist and hand | All |
| NFA | Exploratory procedures on hip and thigh | All |
| NFE | Operations on capsules and ligaments of hip joint | NFE15 |
| NFH | Miscellaneous operations on hip joint (reduction of dislocation) | All |
| NFL | Operations on muscles and tendons of hip and thigh | All |
| NGA | Exploratory procedures on knee and lower leg | All |
| NGD | Operations on meniscus of knee | All |
| NGE | Operations on capsules and ligaments of knee joint | NGE20, NGE25, NGE30, NGE35, NGE40, NGE45, NGE55, NGE60, NGE65 |
| NGH | Miscellaneous operations on knee joint (reduction of dislocation) | All |
| NGL | Operations on muscles and tendons of knee and lower leg | All |
| NHA | Exploratory procedures on ankle and foot | All |
| NHE | Operations on capsules and ligaments of joints of ankle and foot | NHE15, NHE20, NHE25 |
| NHH | Miscellaneous operations on joints of ankle and foot (reduction of dislocation) | All |
| NHL | Operations on muscles and tendons of ankle and foot | All |
